# 

**Published:** 1884-01

**Authors:** 


CONTENTS.

SURGEON GEN'L'S OFFICE LIBRARY. 97407

| | Page. |
|---|---|
| Full Breathing | 1 |
| Ventilation | 2 |
| Scarlet Fever | 3 |
| Causes of Death | 7 |
| Popular Fallacies | 8 |
| Appetite | 9 |
| Throat Ail | 12 |
| How the Stomach Affects the Throat | 14 |

